# Neural Correlates of Modality-Sensitive Deviance Detection in the Audiovisual Oddball Paradigm

**DOI:** 10.3390/brainsci10060328

**Published:** 2020-05-28

**Authors:** Melissa Randazzo, Ryan Priefer, Paul J. Smith, Amanda Nagler, Trey Avery, Karen Froud

**Affiliations:** 1 Department of Communication Sciences and Disorders, Adelphi University, Garden City, NY 11530, USA; rpriefer@adelphi.edu (R.P.); amandanagler@mail.adelphi.edu (A.N.); 2 Neuroscience and Education, Department of Biobehavioral Sciences, Teachers College, Columbia University, New York, NY 10027, USA; pjs2194@tc.columbia.edu (P.J.S.); psa2111@tc.columbia.edu (T.A.); kf2119@tc.columbia.edu (K.F.)

**Keywords:** audiovisual, McGurk effect, fusion, mismatch negativity, N1

## Abstract

The McGurk effect, an incongruent pairing of visual /ga/–acoustic /ba/, creates a fusion illusion /da/ and is the cornerstone of research in audiovisual speech perception. Combination illusions occur given reversal of the input modalities—auditory /ga/-visual /ba/, and percept /bga/. A robust literature shows that fusion illusions in an oddball paradigm evoke a mismatch negativity (MMN) in the auditory cortex, in absence of changes to acoustic stimuli. We compared fusion and combination illusions in a passive oddball paradigm to further examine the influence of visual and auditory aspects of incongruent speech stimuli on the audiovisual MMN. Participants viewed videos under two audiovisual illusion conditions: fusion with visual aspect of the stimulus changing, and combination with auditory aspect of the stimulus changing, as well as two unimodal auditory- and visual-only conditions. Fusion and combination deviants exerted similar influence in generating congruency predictions with significant differences between standards and deviants in the N100 time window. Presence of the MMN in early and late time windows differentiated fusion from combination deviants. When the visual signal changes, a new percept is created, but when the visual is held constant and the auditory changes, the response is suppressed, evoking a later MMN. In alignment with models of predictive processing in audiovisual speech perception, we interpreted our results to indicate that visual information can both predict and suppress auditory speech perception.

## 1. Introduction

The cornerstone of research into the audiovisual nature of speech perception is the McGurk effect, an illusion in which visual articulatory information alters speech perception [1]. The classic McGurk stimulus is an incongruent pairing of visual velar /ga/ dubbed onto the acoustic bilabial /ba/, deriving the fused percept alveolar /da/. Fusion perception has been considered an indicator of audiovisual (AV) integration across a variety of languages and manipulations. One interpretation of the effect of visual articulatory information on speech perception is that as the visual signal naturally precedes the auditory signal, it provides predictive information that constrains the possible identities of the upcoming auditory speech sound [2,3,4,5].

When the McGurk illusion occurs, fusing aspects of conflicting modalities, the derived percept is one that is categorically different from the given input [6]. Fusion responses are dependent upon the ambiguity in the signal, such that the visual aspect of the stimulus overrides the auditory percept. Another type of audiovisual illusion occurs when the input modalities are reversed-auditory /ga/ paired with visual /ba/. Functional magnetic resonance imaging (fMRI) studies utilizing the inverse McGurk stimulus report that participants do not fuse responses into a single percept but rather perceive both sounds simultaneously (/bga/) or only the auditory aspect of the stimulus (/ga/) [1,7,8]. In healthy participants, blood oxygen level dependent (BOLD) responses in the AV integration site, the superior temporal sulcus (STS), are heightened to the inverse McGurk stimuli, auditory /ga/ with visual /ba/ [9]. These responses are likely a product of the maximum salience of both sources of information: bilabial place of articulation and energy of the acoustic burst for the velar phoneme [8,10]. While fused percepts have been extensively studied as an index of integration, few studies have reported on the neural mechanisms of the inverse McGurk stimulus or combination percept.

Processing of incongruent speech may be framed within the literature on predictive processing in sensory perception [11,12,13] and in audiovisual speech perception [2,4,5,14]. Predictive processing accounts of audiovisual speech perception refer to the observations that the visual motion of speech gestures precede the auditory signal by approximately 150 ms in natural speech [15] and that audiovisual integration favors visually-led asynchronies [16,17,18], although a visual lead is not required [19]. The preceding visual signal exerts a constraint on processing by narrowing the possibilities of the upcoming auditory signal. Electrocorticography shows that perceptual accuracy is associated with suppression of the neural response in the superior temporal gyrus (STG) given visual-led as compared to voice-led speech tokens [20]. Arnal (2009) proposed that visual predictability, how well the visual signal predicts the upcoming auditory signal, mediates the functional connectivity between auditory and visual motion areas via a fast, direct corticocortical pathway. The STS, a domain-general integration site, shows enhanced connectivity between visual motion and auditory areas when there is a mismatch between the auditory and visual signals. The mismatch between incongruent auditory and visual signals is mediated by feedback from the STS via a slower and more indirect pathway than that of congruent speech, showing a delayed and enhanced evoked response. In the case of incongruent speech, these feedback loops between STS and auditory and visual areas respectively unfold over a longer time window [14].

### 1.1. Electrophysiological Studies of Audiovisual Fusion

Electrophysiological studies of the McGurk effect have used event-related potentials (ERPs) to index audiovisual integration: the N1 and the P2—early obligatory responses—and the mismatch negativity (MMN)—a change detection response in an oddball paradigm. The N1–P2 complex reflects the influence of visual information on auditory processing at early processing stages [2,3,14,21,22]. When stimuli are presented in an oddball paradigm, the occurrence of the MMN results from the violation of prediction of the upcoming signal [2,23,24,25,26].

#### 1.1.1. The N1–P2 in AV Speech Perception

Processing of auditory speech depends on visual input as early as 100 ms poststimulus onset, as indexed by an amplitude reduction of the N1–P2 event-related potentials in AV compared to auditory-only speech processing. Such crossmodal facilitations depend on the saliency of the visual stimulus (e.g., bilabial place of articulation for /p/ or /b/) and redundancy between the auditory and visual input, regardless of congruency [2]. Quicker identification of AV compared to auditory-only syllables yields a reduced N1 amplitude in the supratemporal auditory cortex [2,17].

Examinations of amplitude suppression of the N1 given incongruent McGurk stimuli compared to congruent AV or auditory-only stimuli suggest that amplitude suppression of the N1 is associated with the strong impact of visual input in resolving incongruency in order to facilitate auditory processing [22]. Since visual saliency is a factor in N1 suppression, phonetic representations can be strengthened or weakened by the content of the visual signal via excitatory or inhibitory mechanisms. Such mechanisms are the result of visually-induced weighting of phonetic representations in the auditory cortex, with N1 suppression when visual input strengthens the illusion [21]. Suppression of the P2, as part of the N1/P2 complex, is typically associated with congruent audiovisual stimuli [27]. One study to date has examined the neural correlates of combination responses and found that the P2 differentiates combinations from fusions in an identification task: P2 suppression for fusion illusions is equivalent to congruent AV speech, while the P2 suppression for combination illusions is greater [22].

#### 1.1.2. The MMN in AV Speech Perception

Previous EEG studies of the McGurk effect have shown a robust mismatch negativity (MMN) ERP response to incongruent audiovisual representations across a variety of manipulations [2,10,23,24,25]. In the oddball paradigm, presentation of the deviant violates sensory expectations, evoking a mismatch response that is manifested as a negative voltage deflection in the ERP elicited in the absence of attention and occurring relatively early poststimulus onset [28,29,30]. In the classic McGurk oddball paradigm, a series of congruent AV syllables (standards) are interspersed with a rare incongruent McGurk syllable (deviant), and perception of the deviants, generally a fused percept, results in a negative voltage deflection in recordings of electrical brain activity [31]. Across studies, the response to the deviant incongruent McGurk stimulus, in which the visual aspect of the stimulus changes and the auditory aspect remains constant, is taken to show that visual speech alters auditory perception in the temporal cortex [32]. The time course of the McGurk MMN is similar to that of the auditory MMN, occurring relatively early, at 150 ms post-auditory onset [25].

Saint Amour et al. (2007) examined the time course and source generators of the McGurk-MMN. They found three distinct phases of McGurk-MMN activity: MMN response at 174 ms poststimulus over the left temporal scalp, remaining left-lateralized to about 250 ms; a secondary phase of activity that spread bilaterally to frontocentral scalp with a maximum amplitude at around 290 ms; and ultimately a third phase peaking at about 375 ms with a return to left-lateralized scalp sites. These phases were subsequently sourced to the temporal lobe posterior to the primary auditory cortex bilaterally, right hemisphere activity in the STG, and two sources of left-hemisphere activity in the transverse gyrus and STG [23].

In summary, previous investigations of the AV MMN elicited by the McGurk effect have shown that it occurs in the absence of acoustic change and is not owing to evoked visual responses. The effect is so robust that when the acoustic stimulus acts as the deviant rather than the visual, the MMN response to McGurk deviants is negated [25]. In terms of predictive processing, the presence of the MMN may be the residual error of a comparison process between incompatible auditory and visual signals [26,33,34]. The visual signal, or viseme, interacts with language-specific phonological representations to constrain possibilities, or create predictions, of the upcoming auditory phoneme. Although visemes represent a constraint on categorization, they are less granular than auditory phonemic representations [3]. Further, they operate on different time scales, with the leading visual articulatory gestures moving more slowly than the rapid acoustic transitions [5]. 

Bhat and colleagues (2015) proposed a dynamic reweighting model, which explains tolerance for temporal asynchrony between auditory and visual signals as a dynamic reweighting of sensory cues [35]. In this dynamic reweighting, when bottom–up complexity or contextual constraints increase, auditory processing shifts from low to high-level networks [36,37,38]. The brain makes use of all available cues to reduce ambiguity, reweighting auditory, visual, or contextual cues via excitatory or inhibitory mechanisms. Under this approach, the visual signal primes the auditory network, creating a new phonetic percept, as in the case of a McGurk fusion [2]. However, the inverse is also theorized: that audiovisual binding may be influenced by shifts in the auditory pathway, as in an inverse McGurk deviant. Inverse McGurk stimuli have not been explored using the MMN paradigm but may provide additional insights into the respective weightings of auditory and visual aspects of the perceptual signal. 

### 1.2. The Present Study

**Figure 1:**
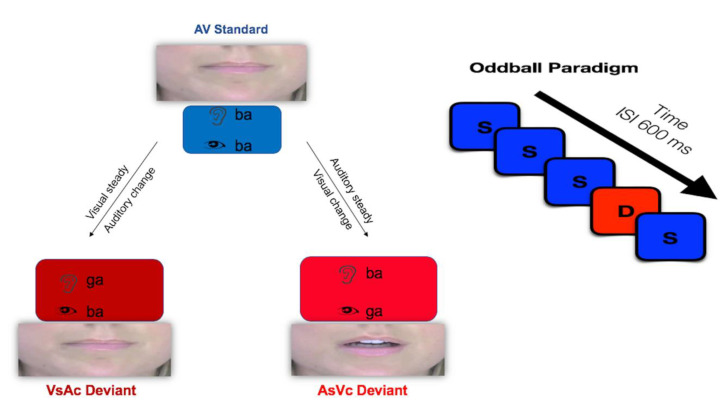
Audiovisual oddball paradigm. In the auditory steady–visual change (AsVc) condition, the visual aspect of the stimulus changes. In the visual steady–auditory change (VsAc) condition, the auditory aspect of the stimulus changes.

The goal of the present study was to examine the neurophysiological correlates of audiovisual conflict under two bimodal conditions. If presentation of incongruent audiovisual stimuli invokes intersensory conflict, then we predicted different neural signatures of conflict processing under conditions in which the auditory or visual aspect of the stimulus changes. Previous studies have examined audiovisual conflict as a function of perception, designating fusion as either similar to congruent stimuli compared to failed illusory percepts or contrasting fused responses to simple congruent stimuli. Here, we employed the classic McGurk stimulus that tends to invoke a fusion response (auditory /ba/, visual /ga/) in contrast to another bimodal condition that typically elicits an audiovisual combination illusion. The combination condition (auditory /ga/, visual /ba/) has been shown to produce a co-articulated merged response (e.g., /bga/) rather than a unique percept. Both the incongruent fusion stimulus and the combination stimulus were juxtaposed against congruent audiovisual syllables (auditory /ba/, visual /ba/) in an oddball paradigm. This design allowed us to examine audiovisual conflict under two different conditions, one in which the visual modality changed (auditory steady–visual change, or AsVc) and one in which the auditory modality changed (visual steady–auditory change, or VsAc). Figure 1 depicts the experimental task in the audiovisual paradigm.

One critique of the body of work utilizing the McGurk effect is that different task demands yield different responses [39]. Oddball paradigms examining the McGurk effect allow for discrimination between stimuli but often require an overt physical response which further modulates brain responses to the stimuli by superimposing attentional or cognitive demands on the perceptual processes. Passive paradigms do not impose a forced choice, but rather, when examining audiovisual fusion, congruency, and conflict, can reflect how the properties of one feature of a stimulus influence perception of the other. Further, passive paradigms that do not require an overt response are ideal for working with aging or impaired populations, such as older adults with presbycusis or adults with aphasia. It is a future goal of this research to develop a passive paradigm to study audiovisual processing in clinical populations in order to further inform diagnostic criteria and evaluation of treatment response. In order to maximize the utility of passive oddball paradigms for studying perception in clinical populations, an adequate control measure is needed to compare with audiovisual McGurk responses and ensure that the MMN response is unique to audiovisual integration. Thus, we examined here in young, healthy adults the use of inverse McGurk stimuli as a comparison measure to the classic McGurk stimulus. 

Aligned with neuroimaging research on predictive processing [2,14,40], we predicted that both AsVc and VsAc deviants would evoke significant differences between congruent audiovisual standards and incongruent deviants in the N1–P2 time windows. N1 amplitudes in response to both AsVc and VsAc deviants were predicted to be more negative than standards, reflecting suppression of redundant information in congruent stimuli. An audiovisual MMN response was predicted for classic McGurk AsVc, signaling an error in predictive processing. We expected that VsAc would not evoke this response, because although categories may merge, the conflicting visual signal should suppress the detection of deviants. Given the prolonged time window of audiovisual feedback loops, we predicted that differences between standards and VsAc deviants may be realized in a later time window post-MMN response. 

Unimodal auditory-only and visual-only responses informed the independence of the bimodal responses. Classic auditory MMN was expected in response to across-category deviants, but visual-only MMNs for speech tokens are more elusive [23,31,41]. In the absence of a visual MMN, when the auditory signal was held constant and the visual signal changed, the resulting MMN would likely not be due to visual perceptual processes. When the visual signal was constant and the auditory changed, evoked auditory responses must override the suppressing visual information.

## 2. Materials and Methods

### 2.1. Participants

Twenty-four participants were recruited for the study. All subjects gave informed consent, and the study protocol (#050517) was approved by the Institutional Review Board of Adelphi University on 22 May 2017. Upon data inspection, five participants were excluded due to <40% usable trials. The remaining 19 participants were young adults (4 males), aged 21 to 35 years (M = 24.05, SD = 3.95). All participants were native English speakers, right-handed, with normal or corrected-to-normal vision and no history of neurological disorders. All participants passed a hearing screening with a threshold of 20 dB from 500 to 2000 Hz. Participants all passed a visual acuity test, the Mars Letter Contrast Sensitivity Test, with a score of 1.52 or better [42]. 

### 2.2. Materials

Stimuli were designed to differ minimally in both modalities. Video tokens were generated by digital recording of a female native American-English speaker saying /ba/ and /ga/. Digital video (Canon Vixia HFR50) and corresponding audio (Blue Mic Yeti Pro, www.bluemic.com) were recorded at a sampling rate of 44.1 KHz and a frame rate of 24 images/second, later trimmed for a total duration of 300 ms per token. The places of articulation for /ba/ and /ga/ differ maximally; thus, the speaker was instructed to open mouth minimally. Jaw aperture was consistent between /ba/ and /ga/ video segments, with a 2.41 and 2.21 cm opening from bottom of upper lip to top of lower lip respectively, as measured in the video frame. This minimal difference is at a threshold that is imperceptible to the human eye [43]. The video frame was cropped using Apple iMovie to reveal only the speaker’s lower face in order to constrain the visual presentation and avoid excess eye movement artifacts during electroencephalographic (EEG) recording. 

The audio tracks were separated from the video and edited in Praat [44] with 50 ms rise/fall to avoid click artifacts in the recording, and amplitudes normalized to 70 dB. Voice onset time between the two tokens differed by 6 ms, 33 ms for /ba/ and 39 ms for /ga/. The vowel segment from one /ba/ recording was removed in Praat, and the spliced /a/ segment was used with the onsets for both /b/ and /g/ so the only difference in the audio was the consonant segment. Also in iMovie, the audio track for /ba/ was dubbed over the video tracks of both /ba/ and /ga/, creating congruent (auditory /ba/, visual /ba/) and incongruent McGurk (auditory /ba/, visual /ga/) AsVc stimuli. Onset of the visual stimulus precedes the acoustic onset by 50 ms, and the acoustics began in the same frame for both tokens. The VsAc condition was similarly created, with an inverse McGurk deviant (auditory /ga/ dubbed onto visual /ba/). Video stimuli are available as Supplementary Materials. Table 1 depicts the stimulus conditions. Participants were presented with a total of 1200 trials, 300 per condition, with 240 standards and 60 deviants in a passive oddball paradigm. Stimuli were pseudorandomized in order to ensure that at least two standards came before every deviant and that deviants were not played consecutively. The interstimulus interval (ISI) for all conditions was 600 ms. Standard /ba/ and deviant /ga/ were also administered in unimodal auditory-only (AO) and visual-only (VO) oddball conditions using the same auditory and visual tracks that composed the bimodal stimuli. Presentation of each condition was counterbalanced across participants.

### 2.3. Procedure

All acquisition of electroencephalographic (EEG) data took place in the Neurocognition of Communication Disorders Laboratory (NCCD Lab) at Adelphi University. EEG data were recorded continuously from 128 electrode sites via a high-impedance amplifier (Electrical Geodesics Incorporated, Eugene, OR, USA) throughout the experiment in a sound shielded room. HydroCel Geodesic Sensor Nets [45] were selected based on head circumference, soaked in a potassium chloride solution, and fitted for alignment at the vertex. After application of the sensor net, individual electrodes were adjusted to maintain impedance thresholds below 40 kΩ. Horizontal eye movements and saccades were measured at channels located at positions around the orbital and brow bones. Amplified analog voltages were digitized at a 500 Hz sampling rate. EEG data were referenced to the vertex electrode during recording and later re-referenced offline to the average reference. 

Participants were seated approximately 85 cm from computer monitor and instructed to relax and face the screen. The stimuli were presented at 25% of the screen size, subtending a 28 degree visual angle. Participants were told they would see and hear some speech sounds but that they did not have to focus intently on the computer monitor or the sounds.

### 2.4. EEG Preprocessing and Analysis

Raw EEG data were preprocessed using NetStation, version 5.4.2, (Electrical Geodesics, Inc., Eugene, OR, USA). Raw data were digitally filtered offline with a 1–45 Hz bandpass filter and artifacts were automatically rejected using protocols for the removal of movement and physiological artifacts. Bad channels (defined as >150 μV), blink artifacts (defined as >140 μV), and eye saccades (defined as >55 μV) were removed using an in-house NetStation script. Data were re-referenced to the average reference, which was calculated by subtracting the mean of all electrodes from each individual channel. 

The ERP waveform was segmented into 700 ms epochs including a 100 ms prestimulus baseline and a 600 ms poststimulus period. For bimodal stimuli, epochs were segmented at the onset of the visual stimulus of the video, which led the acoustic track by 50 ms. Hence, the 0–50 ms time window represents only visual processing, while beyond 50 ms represents bimodal presentation. Individual segments were baseline corrected using the 100 ms prestimulus period, thus removing baseline unimodal visual activity from the poststimulus period in the bimodal stimuli [23]. Each segment was manually inspected for artifacts and bad channels and rejected accordingly. Only segments free of artifacts were included in ERP averages for each condition. Following preprocessing in NetStation, data files were exported to R version 3.4.4 and analyzed using in-house scripts to derive peak latencies and adaptive mean amplitudes in electrode montages of interest. These included channel groups corresponding to established sites of auditory, audiovisual, and visual speech processing, including frontal, left temporal, and central parietal channels [23,24,31,46,47,48,49,50,51]. Consistent with previous audiovisual oddball studies, adaptive means and peak latencies were calculated in 4 time windows of interest corresponding to the N1 (50–100 ms), P2 (100–150 ms), early MMN (150–200 ms), and late MMN (300–400 ms) [2,24,25,52]. Peak latencies were defined as the time of maximum voltage in time windows of interest, and adaptive means were calculated for each participant by averaging 10 samples (20 ms) on either side of the peak.

For each time window of interest, two-way repeated-measures ANOVAs (3 × 2 × 2) with factors montage (frontal, left temporal, central parietal), condition (fusion, combination), and stimulus type (standard, deviant) were carried out for responses to each bimodal condition separately (AsVc and VsAc). Since presence of the MMN was the main focus of the study, planned comparisons were carried out where a significant main effect of stimulus was found. In this study, MMN is defined as a significant difference between deviant and standard (deviant < standard, *p* < 0.05). For characterization, unimodal AO and VO conditions were analyzed for presence of N1, P2, MMN, and late negativity in montages of interest. Factors related to electrode montage were only of interest if they interacted with stimulus effects, so main effects of montage are not reported here. 

## 3. Results

### 3.1. Usable Trials

Following data pre- and postprocessing, numbers of usable trials were calculated for each participant based on stimulus type in each condition. The threshold for inclusion based on usable trials per condition was set at 40%. ANOVA was carried out to ensure that there were no differences in numbers of usable trials by condition. There were no significant differences in the number of usable responses to standards across conditions (F (3, 72) = 0.10, *p* = 0.960, η^2^ = 0.004), with means and standard deviations as follows: AsVc (M = 201, SD = 59.1), VsAc (M = 202, SD = 51.8), auditory only (M = 200, SD = 72.7), and visual only (M = 192, SD = 65.2). There were likewise no significant differences in the number of usable responses to deviants across conditions (F (3,72) = 0.95, *p* = 0.42, η^2^ = 0.038), with means and standard deviations as follows: AsVc (M = 49, SD = 15), VsAc (M = 49, SD = 14.3), auditory only (M = 47, SD = 8.91), and visual only (M = 45.3, SD = 8.93). 

### 3.2. ERP Results

Difference waves are depicted for visualization purposes below, but all analyses and planned comparisons were conducted over stimulus response waves by condition. Table 2 depicts a summary of event-related potential (ERP) effects.

#### 3.2.1. Unimodal Auditory Results

N1 (50–100 ms)

No significant main effect of stimulus (*p* = 0.34) or interaction between stimulus and montage (*p* = 0.33) was found. 

P2 (100–150 ms)

No significant main effect of stimulus (*p* = 0.13) or interaction between stimulus and montage (*p* = 0.27) was found.

Early MMN (150–200 ms)

Repeated-measures ANOVA revealed a significant main effect of stimulus (F (1, 18) = 6.74, *p* < 0.05, η_p_^2^ = 0.272), with significant MMN (response to deviant more negative than standard) in the left temporal montage (*p* < 0.05), as revealed by planned comparisons. No interaction between stimulus and montage (*p* = 0.73) was found. 

Late MMN (300–400 ms)

**Figure 2:**
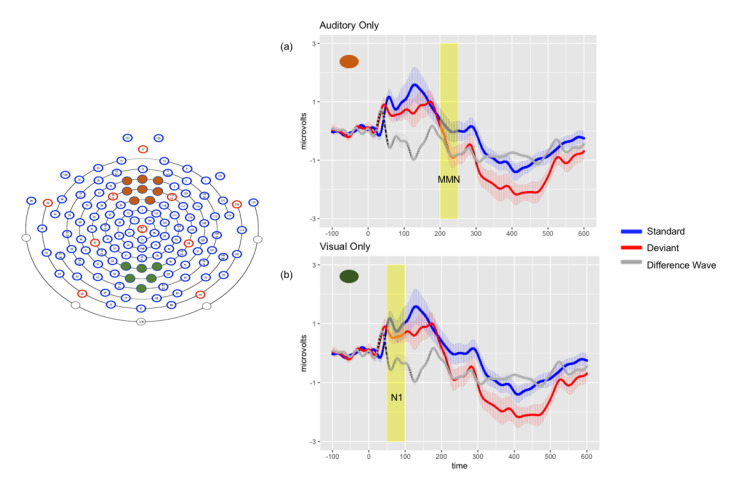
Left: Electrode montages for auditory only (orange) and visual only (green) grand average waveforms. Right: Grand average waveforms for auditory only (Panel **a**) and visual only (Panel **b**) conditions. Solid lines represent the average response in microvolts across time in milliseconds, shaded lines represent the standard error of the means at each time point, and shaded rectangle represents time window of significance.

Repeated-measures ANOVA revealed a significant main effect of stimulus (F (1, 18) = 4.82, *p* < 0.05, η_p_^2^ = 0.211), with responses to deviant stimuli again more negative than to standards. There was a significant interaction between stimulus and montage (F (2, 36) = 8.16, *p* < 0.01, η_p_^2^ = 0.312), driven by significantly greater negativities in response to the deviants in all montages (*p* < 0.001). Planned comparisons revealed a significant late negativity in the frontal montage (*p* < 0.001), shown in Figure 2.

#### 3.2.2. Unimodal Visual Results

N1 (50–100 ms)

There was no main effect of stimulus (*p* = 0.174), but a significant interaction between stimulus and montage was found (*p* < 0.001), driven by significant N1 differences with responses to deviants more negative than standards in left temporal (*p* < 0.05) and central parietal (*p* < 0.01) montages. Significant differences were found in the frontal montage, but with standard more negative than deviant (*p* < 0.001). Figure 2 depicts the significant N1 observed in the central parietal montage.

P2 (100–150 ms)

There was no significant main effect of stimulus (*p* = 0.64) or interaction between stimulus and montage (*p* = 0.15).

Early MMN (150–200 ms)

ANOVA revealed significant main effects of stimulus (F (1, 18) = 16.78, *p* < 0.01, η_p_^2^ = 0.281) and of montage (F (2, 36) = 48.63, *p* < 0.001, η_p_^2^ = 0.730). There was a significant interaction between stimulus and montage (F (2, 36) = 18.74, *p* < 0.001, η_p_^2^ = 0.510). Planned comparisons revealed significant differences between standards and deviants in the frontal (*p* < 0.001) and central parietal montages (*p* < 0.001), but only a marginally significant difference in the left temporal montage (*p* = 0.06). However, all differences were associated with greater negativities in response to standard than deviant stimuli. 

Late MMN (300–400 ms)

No main effects of stimulus (*p* = 0.78) or montage (*p* = 0.54) were found in the late time window for the visual condition. 

#### 3.2.3. Audiovisual Results

N1 (50–100 ms)

Repeated-measures ANOVA revealed a significant interaction between condition and montage (F (2, 36) = 10.48, *p* < 0.001, η_p_^2^ = 0.368) and between stimulus and montage (F (1, 18) = 9.89, *p* < 0.001, η_p_^2^ = 0.355) but no main effect of stimulus (*p* = 0.07) or condition (*p* = 0.75). Bonferroni-adjusted posthoc pairwise comparisons revealed significant differences between the VsAc condition in each montage: frontal vs. left temporal (*p* < 0.001), frontal vs. central parietal (*p* < 0.001), and left temporal vs. central parietal (*p* < 0.05). There were significant differences between responses to the AsVc condition in frontal vs. left temporal (*p* < 0.001) and frontal vs. central parietal (*p* < 0.001) montages. Planned comparisons between standards and deviants for each condition in each montage confirmed a significant N1 (deviant more negative than standard) response to the VsAc condition in the left temporal montage (t (18) = −2.19, *p* < 0.05), and to the AsVc condition in both left temporal (t (18) = −2.81, *p* < 0.05) and central parietal montages (t (18) = −3.72, *p* < 0.01). 

P2 (100–150 ms)

Repeated-measures ANOVA revealed significant main effects of condition (F (1, 18) = 5.57, *p* < 0.05, η_p_^2^ = 0.236) but no main effect of stimulus (*p* = 0.89).

Early MMN (150–200 ms)

Repeated-measures ANOVA revealed a significant main effect of condition (F (1, 18) = 10.44, *p* < 0.01, η_p_^2^ = 0.367) and of stimulus (F (1, 18) = 14.70, *p* < 0.001, η_p_^2^ = 0.450). Significant interaction effects were found for condition by stimulus (F (1, 18) = 4.65, *p* < 0.05, η_p_^2^ = 0.205), condition by montage (F (2, 36) = 7.55, *p* < 0.01, η_p_^2^ = 0.295), and stimulus by montage (F (2, 36) = 3.47, *p* < 0.001, η_p_^2^ = 0.313). Responses to the VsAc and AsVc conditions differed from each other in the central parietal montage (*p* < 0.01), but not the frontal (*p* = 0.27) or left temporal montages (*p* = 1.00). Planned comparisons confirmed that these effects were driven by a significant MMN (deviant more negative than standard) response to the AsVc condition in the left temporal montage (t (18) = −3.45, *p* < 0.01) and in the central parietal montage (t (18) = −3.49, *p* < 0.01).

Late MMN(300–400 ms)

**Figure 3:**
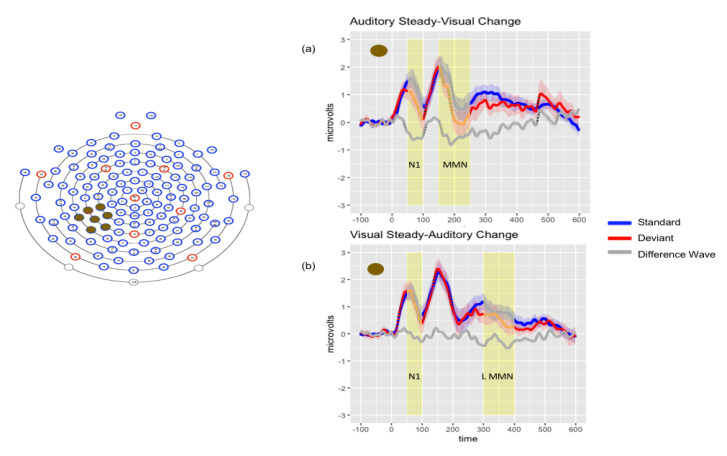
Left: Electrode montage for grand average waveforms of audiovisual conditions. Right: Grand average waveforms for AsVc (Panel **a**) and VsAc (Panel **b**) conditions. Solid lines represent the average response in microvolts across time in milliseconds; shaded lines represent the standard error of the means at each time point.

Repeated-measures ANOVA revealed a significant main effect of stimulus (F (1, 18) = 21.52, *p* < 0.001, η_p_^2^ = 0.545). Planned comparisons confirmed that these effects were driven by a significant negativity (deviant more negative than standard) in response to the VsAc condition in the left temporal (t (18) = −3.28, *p* < 0.01) and central parietal (t (18) = −2.41, *p* < 0.05) montages. Figure 3 depicts audiovisual results for AsVc and VsAc conditions.

#### 3.2.4. Posthoc Analyses

Due to the differential findings of significant negativities in response to the AsVc deviants in the 150–200 ms time window and the VsAc deviants in the 300–400 ms time window in the left temporal and central parietal montages, we wanted to further characterize these effects. We cast a wide temporal net to examine peak latency for AsVc and VsAc in the period of 150–400 ms over left temporal and central parietal montages. Peak latency was 262 ms for AsVc deviant responses and 299 ms for VsAc deviant responses in the left temporal montage; 322 ms for the AsVc deviant responses and 340 ms for the VsAc deviant responses in the central parietal montage. In the left temporal montage, repeated-measures ANOVA revealed a main effect of condition (F (1, 18) = 8.96, *p* < 0.01, η_p_^2^ = 0.332, M_diff_ = 30.5 ms), indicating that the peak latency was significantly later in response to VsAc deviants. No significant main effects of condition (*p* = 0.354) or stimulus (*p* = 0.415) were found in the central parietal montage. A similar analysis of N1 peak latency differences revealed no significant effects. 

## 4. Discussion

Speech perception is an inherently multisensory process that integrates the visual perception of the movements and expressions of the speaker with the acoustic signal of speech. As exemplified by the McGurk effect, in speech perception, the visual stimulus overrides the auditory stimulus, thus changing the percept altogether [1,6]. We used an audiovisual oddball paradigm to examine differential responses to deviants elicited by changing either the auditory or visual aspect of the stimulus. Stimuli were designed to be minimally different in both auditory and visual domains. We found significant differences between standards and deviants for both bimodal conditions in the N1 time window, and an early MMN for AsVc, and a late MMN for VsAc. A significant N1 was found for the visual-only condition, and a significant MMN was present for the auditory-only condition.

### 4.1. N1–P2

A significant N1 was present in response to both types of AV deviants, over left temporal electrodes for both conditions, and over central parietal electrodes for AsVc. Previous studies indicate that suppression of the N1 (e.g., amplitude reduction) is associated with redundancy between visual and auditory signals [2,21,40,53]. In the passive oddball paradigm, the congruency of the auditory and visual signals of the standard bimodal stimulus suppressed the amplitude of the N1 relative to the incongruent deviant in both conditions. There was no effect of condition in the N1 time window, indicating that both deviants exert similar influence in generating congruency predictions, regardless of which modality changes. While latency facilitation at the N100 is well-established, the literature regarding amplitude suppression at the N100 is less clear [20]. Amplitude suppression at the N1 has typically been studied by comparing unimodal auditory-only responses to audiovisual responses. In the case of a comparison between congruent and incongruent bimodal stimuli, we found that the congruent standards are suppressed compared to the incongruent deviants, regardless of the modality driving the deviance. Arnal et al. (2009) [10] theorized that amplitude reduction in the case of the M100 may be relative to the ambiguity of the viseme in constraining phonological predictions. On this view, amplitude suppression of responses to congruent relative to incongruent bimodal stimuli occurs via crossmodal inhibition of phonetic representations in auditory cortex driven by constraining visual input. The early occurrence of this process, undifferentiated by modality of the deviance, is aligned with a direct, fast visual-to-auditory route [14]. If this is correct, then the presence of a significant N100 response to the visual-only condition over left temporal and central parietal electrodes could similarly reflect deviance detection due to change in the visual modality. However, we found significant differences between standards and deviants in both bimodal conditions, irrespective of a visual or auditory change.

The audiovisual N1 and P2 are typically discussed as an early complex indexing visually-induced latency facilitation and amplitude suppression. However, these two components may be experimentally dissociable [54]. In contrast to previous studies, we did not find differences between responses to incongruent AsVc and VsAc deviants compared to congruent standards in the P2 time window [22]. One reason may be task differences, since our paradigm was a passive oddball rather than an identification paradigm. In the context of an identification task, P2 components may be related to attention and classification, which was not relevant to our paradigm [55]. Additionally, the P2 is believed to encode differences in basic acoustic features such as voice onset time, which were minimized in the design of our unimodal stimuli (the small difference in VOT and use of the same vowel token, in addition to normalizing amplitude, duration, and pitch, reduces differences between the acoustic features of each token). This may have flattened perceptual differences of the acoustic stimulus properties in the generation of bimodal stimuli [56]. 

### 4.2. Early and Late MMN

While both deviants produced a significant difference from standards at the N1, presence of the MMN in the early and late time window differentiated the AsVc from VsAc deviants. It is theorized that the MMN is driven by a failure to predict bottom–up input that suppresses prediction error [2]. In our audiovisual oddball paradigm, we manipulated the modality of the deviant in order to examine how audiovisual interactions are influenced by the content of each sensory modality. In the AsVc, the prediction error is generated by a change to the visual percept while the auditory stimulus is held constant, while in the VsAc condition, the prediction error is generated by a change to the auditory percept while the visual stimulus is held constant.

As predicted, the audiovisual MMN for AsVc stimuli was present. The visual change evokes a mismatch response by changing the auditory percept. This robust effect has been found consistently and crosslinguistically across a range of stimuli in both electroencephalography and magnetoencephalography [2,23,24,25,31,32,41]. In the current study, the MMN in the AsVc condition was found over the left temporal electrodes, consistent with studies of the source analysis of the left-laterialized phonetic-specific McGurk MMN in the transverse gyrus and the superior temporal gyrus [23]. In our AsVc condition, the auditory stimulus is held constant yet generates a phonetic mismatch. In contrast, the auditory-only conditions showed a frontal MMN and no MMN was present for the visual-only condition. The lack of MMN in the visual-only condition, and the baseline subtraction of evoked visual activity, indicate that the AsVc MMN over left temporal scalp is induced by visual influence on the auditory percept. 

We interpret the effects of the VsAc condition in light of theories of predictive processing in audiovisual speech perception. Here, we propose that the constant visual standard suppressed the MMN, resulting in a later negativity. To our knowledge, this stimulus which typically induces combination illusions has not been explored in the audiovisual oddball paradigm. In our VsAc condition, visual aspects of the stimulus are held constant, while acoustic aspects change. In the auditory-only condition, a frontal MMN response was observed. In the VsAc condition, the MMN occurred in a later time window, later than similar responses in both auditory and AsVc conditions. The topography of the VsAc MMN was the same as in the AsVc condition, over left temporal scalp electrodes, while the acoustic MMN presented over the frontal electrode montage. Posterior MMN is presumed to be associated with processing of phonetic rather than acoustic changes [51,57]. In natural speech, the visual onset of articulatory gestures constrains predictions of the upcoming auditory signal, and visual information transfers caudorostrally through STG before the onset of the acoustic burst [58]. Our VsAc stimuli represent maximum salience of the visual and acoustic signals, both the bilabial articulatory gesture and the acoustic velar burst. The visual lead inhibits representations of incompatible phonemes [20]. In contrast to effects seen at the N100 in this study, and N100/M100 in others [14,59], which indicate an early, fast, and direct corticocortical connection between auditory and visual sensory areas, effects in the MMN time window may occur via a slower, indirect feedback pathway whereby STS inhibits auditory and visual areas. Given two incongruent conditions, there may be a time gradient aspect to the mismatch effect in these feedback loops, such that fusion illusions typically associated with these stimuli occur earlier than the types of combination illusions typically associated with our VsAc stimulus. We propose that in the VsAc condition, visual predictions constrain and suppress the earlier, automatic response that was seen in the AsVc and auditory-only conditions, and a process of comparison (requiring classificatory mechanisms) as reflected by the MMN in the later time window. 

In summary, we interpret the time course and directionality of our effects in terms of recent models that build upon the predictive coding hypothesis [3,4,14,40]. Amplitude reduction of responses to congruent standards compared to deviants occurs early in the N100 time window via the fast, direct corticocortical pathway. Although responses to congruent standards are suppressed compared to responses to incongruent deviants, no amplitude differences are found between the two types of incongruent stimuli at this stage, regardless of modality change. Subsequently, a slower, indirect feedback pathway via the STS signals mismatch between visual prediction and auditory input, such that the prediction error is generated in the MMN time window. This occurs earlier for the AsVc condition, where the visual signal changes, presumed to create a new fusion percept; however, this response is further suppressed by the visual constant in the VsAc condition when the auditory changes, so that the MMN response is later for this condition. 

### 4.3. Modality-Sensitive Illusory Perception

Morís Fernández et al. demonstrated that the resolution of intersensory conflict between auditory and visual modalities is critical for the McGurk illusion to occur [60]. Conflict detection and subsequent resolution, served by the anterior cingulate cortex (ACC) and left inferior frontal gyrus (lIFG), results in the McGurk fusion. We argue that early detection of conflict (incongruency) is reflected at the N100, which was equivalent for both types of deviants. Resolution of the incongruency conflict at a later processing stage is reflected by the early MMN for AsVc, deviants in which a new, fused percept is typically generated. VsAc deviants induce a prolonged process of conflict resolution due to suppression via the visual modality, resulting in a late MMN. 

Neural representations of the fusion illusion in human parabelt auditory cortex correlate with the neural representation of the visual stimulus, rather than with the acoustic stimulus [58]. Illusion perception is biased toward the strongest cue, typically the visual [40]. This perceptual bias is theorized to result from inhibition of low-level acoustic features, while strengthening connections to higher-level networks for more complex feature extraction. On the other hand, illusion failure is presumed to be biased toward the auditory cue [21]. With inverse McGurk or combination stimuli, associated with our VsAc stimulus, intermediate percepts are relayed by two phonemes, creating unstable representations, which perhaps are not biased toward either modality, resulting in perception of both. Abbott and Shahin (2018) accidentally induced combination percepts in their stimuli by manipulating early formants of /ba/ and /wa/ (inducing /bwa/ and /wba/) to make them perceptually unstable and more susceptible to visual influence [53]. It is unclear whether combination conditions therefore require conflict to be resolved, by analogy with fusion processing, or whether the illusory percepts generated under these conditions reflect a different response. Our results indicate that prediction error generated by VsAc deviants, typically associated with combination illusions, is temporally suppressed by preceding visual information, which may create a protracted time window for perceptual resolution, allowing more leeway for temporal integration rather than overriding both acoustic and visual inputs to produce a fusion. This same protracted time course of processing also permits reanalysis, which results in the inherent representational instability of the combination percepts, while the shorter time to classificatory responding (indexed by the early MMN) precludes such instability for fusions. 

### 4.4. Limitations and Future Directions

A longstanding critique of the McGurk effect, or other manipulations of the illusion, is that incongruent speech tokens are not an ecologically valid measure of the uptake of visual information to support speech perception. Indeed, finding correspondence between speech reading skill and the McGurk effect has not been successful [56,61,62,63]. Alsius (2018) discusses some of the problematic aspects of using the incongruent McGurk stimulus as a proxy for AV integration. Incongruent AV speech stimuli are not behaviorally equivalent to congruent AV speech stimuli. They are poor category exemplars, generate longer reaction times, are more susceptible to image degradation, and are more vulnerable to spatial or temporal adjustments compared to congruent AV stimuli [45] (see Alsius 2018 for review). Lesion studies suggest that audiovisual fusion does not provide a measure of the ability to extract visual information from bimodal stimuli [64]. Nonetheless, resolution of intersensory conflict via the McGurk effect remains the prevailing index of audiovisual integration in the literature. 

The current study employed a passive oddball paradigm to examine the weighting of auditory and visual modalities in deviance detection. In this paradigm, the perception of one aspect of the stimulus is expected to influence other aspects of the stimulus, ideal for examination of the dynamics of audiovisual integration. Indeed, our findings suggest that the McGurk effect in an oddball paradigm may index a weighting of cues from different modalities, rather than integration per se. However, with this paradigm, we do not have behavioral data to compare with the evoked responses, and therefore, we cannot say for sure how the deviants were perceived. Likely, there was individual variability within and between participants in the perception of the modality changes in the deviants [7,61,65]. Future studies should further address the categorization and identification of percepts under fusion and combination conditions. Still, the passive oddball paradigm provided the most effective means for this investigation of pre-conscious categorization responses to multisensory speech percepts. 

The auditory MMN has been hailed as a clinically useful tool for understanding central auditory processing across different clinical populations, as well as recovery from neurological insult. However, the audiovisual MMN, along with its implications for understanding ongoing integrative perception of our complex and multisensory environment, has been underexplored in clinical contexts. We argue that in young, healthy adults, auditory deviance detection can be temporally suppressed by conflicting visual information. In clinical populations, the VsAc condition may provide a comparison against classic McGurk fusions as a measure of the strength of audiovisual interactions during speech processing. 

## 5. Conclusions

The results of this study indicate that audiovisual deviance detection is modality-sensitive. Although early obligatory responses are essentially equivalent for stimuli associated with fusion and combination illusions, responses to false predictions generated by the different types of incongruent stimuli differ and are reflected by the MMN in early and late time windows. Manipulation of the modality of the deviant has consequences for the predictions generated by the standard stimuli, with mismatch responses to the deviants occurring in different time windows. When the auditory information is held constant, and the visual changes, the prediction error is generated earlier, resulting in a visual facilitation of multisensory processing. Conversely, when the visual is held constant and the auditory changes, visual information suppresses the multisensory resolution of conflicting input. Hence, our findings support the hypothesis that visual information can both suppress and predict auditory information, underscoring the importance of multisensory integration in speech perception.

## Figures and Tables

**Table 1:** Schematic of standards and deviants in each condition in the passive oddball paradigm: auditory steady–visual change (AsVc); visual steady–auditory change (VsAc); auditory-only (AO); and visual-only (VO).

Condition	Standard 80%	Deviant 20%
**AsVc**	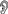 /ba/ + 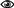 /ba/	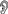 /ba/ + 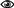 /ga/
**VsAc**	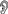 /ba/ + 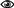 /ba/	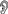 /ga/ + 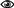 /ba/
**AO**	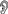 /ba/	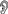 /ga/
**VO**	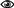 /ba/	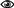 /ga/

**Table 2:** Summary of significant event-related potential (ERP) effects where deviant is more negative than standard by condition and time window (stimulus type and montage specified by cell). M_diff_ = mean difference between deviant and standard; VsAc = Visual steady, Auditory change; AsVc = Auditory steady, Visual change; FR = frontal montage; LT = left temporal montage; CP = central parietal montage. * *p* < 0.05, ** *p* < 0.01, *** *p* < 0.001.

		CONDITION			
		Unimodal		Bimodal	
		Auditory Only M_diff_ μV	Visual Only M_diff_ μV	VsAc M_diff_ μV	AsVc M_diff_ μV
Time Window	N1 (50–100 ms)	n.s.	−0.430 ** LT −1.096 ** CP	−0.312 * LT	−0.531 ** LT −0.654 ** CP
	P2(100–150 ms)	n.s.	n.s.	n.s.	n.s.
	Early MMN (150–200 ms)	−0.451 * LT	n.s.	n.s.	−0.699 ** LT −1.09 ** CP
	Late MMN (300–400 ms	−1.119 *** FR	n.s.	−0.4756 ** LT −0.403 * CP	n.s.

## Supplementary Materials

The following are available online at https://www.mdpi.com/2076-3425/10/6/328/s1, Video S1: Standard congruent /ba/ stimulus, Video S2: Incongruent fusion (McGurk stimulus), Video S3: Incongruent combination stimulus.
